# Round about the Asylums.—V

**Published:** 1888-04-21

**Authors:** 


### Round About the Asylums.—V.

By a Medical Superintendent.

### HEREFORD COUNTY ASYLUM.

From this, the sixteenth annual report, we learn that the asylum contained 357 patients at the end of the year, and practically they all belonged to the county and city of Hereford. Want of accommodation was much felt, and instead of anticipating this want by adding to the asylum, and so retaining their private patients, the visitors have adopted the policy of sending twenty-two chronic cases to the workhouses, and discharging nearly all their private patients. Thus they have postponed the evil day; but that they have thereby obviated the necessity for building cannot be even dreamt of. Cases transferred to workhouses almost invariably go back in mental and bodily condition, and it is questionable whether the step should ever be advised. The weekly rate is 8*s.* 3*d.*, and the capitation grant reduces this to 4*s.* 3*d.*, as far as the guardians are concerned. The patients will cost close on 6*s.* in the workhouse; so that there is a loss rather than a gain to the local rates. In the meantime the profits on the private patients have been thrown away, and the necessity for building not prevented, but only postponed. The only truly economical course for visitors to pursue is to keep their accommodation in excess of their requirements. If this be not done, all asylum history shows that it will fall below it; and the inevitable results are the discharge of paying patients and the entering into expensive contracts with other asylums. The recovery rate is above the average here, and the death rate below it.

### THE FRIENDS' RETREAT, YORK.

When an institution for the insane publishes its ninety-first annual report, it must have seen in its lifetime many wonderful changes in the treatment of its patients; but this is perhaps less true of the Retreat than it would be of any other old asylum, not because it has not kept abreast of the best of the hospitals, but because it adopted the principles of humane treatment at the beginning of its career. Thus it had not so much to change. It now contains about 150 patients, and there are three medical officers. This is a higher proportion than even the American asylums can boast of, and it must afford the most ample time for the careful treatment of individual cases, and for the scientific investigation of cloudy points connected with insanity. The weekly cost of maintenance was 35*s.* 10*d.*, and the average charge was 41*s.* 2*d.* The recoveries were 29·76 per cent. on the admissions, and the deaths 8 per cent. on the average number resident. Unlike some hospitals, it conceals neither its charges nor its cost; and it does not close its doors to epileptics and paralytics. Moreover, it does not limit its good work to members of the Society of Friends. Nearly one-half cf the patients are of other denominations. A convalescent home is permanently kept up at Scarborough. This is a much better plan than the common one of engaging temporary lodgings at some seaside place. The Commissioners' report is conspicuous by its absence.

### LEICESTER BOROUGH ASYLUM.

The visitors' report is somewhat longer than usual, and deals with many matters. It would seem that 157 of the Derby borough patients are boarded out in this asylum, and have been for years, so that the Leicester people must have reaped a nice harvest of not less than £1,500 a-year in profits —a state of things somewhat more satisfactory to the ratepayers than that which we had to notice of the Hereford Asylum. The asylum contains 483 patients, and the weekly rate of maintenance is 10*s.*; 42 per cent. of the admissions were discharged recovered, and the death rate was 10·43 on the average number resident. Thirteen of the deaths were due to brain diseases, four to phthisis, and six to pneumonia. It is curious how fatal the last-named disease is to lunatics. Post-mortem examinations were made in every case of death. There were several attempts at suicide, but fortunately no attempt was successful. One man swallowed four penny pieces. The insane are extremely fond of hoarding up trifles, and it does not appear whether this man wanted to put his money in a safe place or to commit suicide. The Jubilee was celebrated here in good style on the 21st June, and many other entertainments were given during the year. The farm produced £305 profit; but "no rent" is the system pursued here also, and we are not enlightened about the prices allowed to the farm for goods supplied to the asylum. There is a fine simplicity about this style of farming that must commend itself to every practical agriculturist.

### NORTH WALES ASYLUM AT DENBIGH.

Very important questions are discussed in this report. The accommodation is not nearly sufficient for the wants of the counties to which the asylum belongs, namely—Denbigh, Flint, Anglesey, Carnarvon, and Merioneth; and the deficiency would seem to be chiefly on the female side of the house. Twenty-five patients have been removed to the Abergavenny Asylum; and, exclusive of these, there are 249 female patients in the asylum, 87 of whom occupy wards which were intended for the men. The asylum is forty years old, and hence is under the old measurements as regards the cubic space allotted to each patient. If this were to be altered to the new requirements, which no one can say are one whit too high, the alteration would reduce the accommodation to 151 beds on the women's side, being not very much more than half a bed-space to each patient. The visitors talk of adding 200 beds to the house, with suitable enlargements of the laundry, kitchen, stores, &c., and they estimate the expense roughly at £20,000. This sum, *properly expended*, would be more than sufficient to provide all that is wanted. Additions to asylums ought to be built, and indeed have been built, for £75 per bed. The want of space is a long-standing deficiency in the Denbigh Asylum, and the Commissioners in Lunacy animadvert somewhat strongly on it in their last report. It is, however, easy to understand the feeling of the committee about spending public money at such a time of depression as this is, and much may be forgiven them on this score. Nevertheless, it is certain that the money must be spent sooner or later, and the earlier the accommodation is provided the better it will be for the ratepayers and the patients too. In the meantime, twenty-five patients are boarded out, and the difference between the cost at Denbigh and the charge at Abergavenny will amount to nearly £150 a-year. This the ratepayers have to find until additions are built. The Commissioners also complain of the insufficiency of the staff, and the committee have appointed three additional attendants. On referring to the detailed statement of the weekly cost we find that "salaries and wages" figure at 1*s.* 9¼*d.* Wages are doubtless lower in North Wales than in most parts of England; still we doubt whether in an asylum the size of Denbigh an efficient staff, numerically and otherwise, can be kept up for 1*s.* 9¼*d.* a-week per patient, and we trust next year to find it has risen to the average in English asylums, namely, 2*s.* 4¼*d.* We note that the Medical Superintendent has only £450 a-year. The weekly rate is 7*s.* 9¼*d.* Private patients pay from 10*s.* 6*d.* to £3 3*s.* a-week. We should like to know what the latter receive for this sum. The recoveries were 29·53 on the admissions, and the deaths were 9·12 on the average number resident. A good many minor improvements have been carried out during the year, and among these we notice the artificial heating of the hall and some of the single-bedded rooms, improved sanitary arrangements, replanting of airing courts, etc. The Commissioners remark that Dr. Cox "is evidently anxious to mitigate, as far as possible, the evils arising from the many defects in this asylum." In the farm accounts rent is included, and it appears that £141 was realised as profit. Provisions are stated to be reckoned at market value. Does this mean the price which the asylum could buy or sell in the market? Selling and buying are different things.

